# Temperatures and hypolimnetic oxygen in German lakes: Observations, future trends and adaptation potential

**DOI:** 10.1007/s13280-024-02046-z

**Published:** 2024-07-05

**Authors:** Robert Schwefel, Lipa G. T. Nkwalale, Sylvia Jordan, Karsten Rinke, Michael Hupfer

**Affiliations:** 1 Department of Ecohydrology and Biogeochemistry, Leibniz-Institute of Freshwater Ecology and Inland Fisheries, Müggelseedamm 301, 12587 Berlin, Germany; 2 Department of Lake Research, Helmholtz Centre for Environmental Research – UFZ, Brückstr. 3a, 39114 Magdeburg, Germany; 3 Department of Aquatic Ecology, Brandenburg University of Technology Cottbus-Senftenberg, Seestraße 45, 15526 Bad Saarow, Germany

**Keywords:** Climate change, Lake eutrophication, Lake stratification, Lake temperatures, Numerical modelling, Oxygen

## Abstract

We investigated trends in temperature, stratification, and hypolimnetic oxygen concentration of German lakes under climate change using observational data and hydrodynamic modelling. Observations from 46 lakes revealed that annually averaged surface temperatures increased by + 0.5 °C between 1990 and 2020 while bottom temperatures remained almost constant. Modelling of 12 lakes predicted further increases in surface temperatures by 0.3 °C/decade until the year 2099 in the most pessimistic emission scenario RCP 8.5 (RCP 4.5: + 0.18 °C/decade; RCP 2.6: + 0.04 °C/decade). Again, bottom temperatures increased much less while summer stratification extended by up to 38 days. Using a simplified oxygen model, we showed that hypolimnetic oxygen concentrations decreased by 0.7–1.9 mg L^−1^ in response to the extended stratification period. However, model runs assuming lower productivity (e. g. through nutrient reduction) resulted in increased oxygen concentrations even in the most pessimistic emission scenario. Our results suggest that the negative effects of climate change on the oxygen budget of lakes can be efficiently mitigated by nutrient control.

**Supplementary Information:**

The online version contains supplementary material available at 10.1007/s13280-024-02046-z.

## Introduction

Climate change is one of the biggest challenges of the twenty-first century and heavily affects the physical conditions and ecosystems of lakes (Woolway et al. 2020). Atmospheric warming is increasing surface temperature and heat content, strengthening and prolonging lake stratification, and consequently impacting ecological parameters on all trophic levels from phytoplankton growth (Anneville et al. 2002) to fish habitats (Jeppesen et al. 2014; Kraemer et al. 2015). Hence, lakes will undergo notable structural, functional, and dynamic changes (Mi et al. 2020), necessitating effective adaptation strategies (Bartlett and Dedekorkut-Howes 2022; Wang et al. 2023) to mitigate the effects of climate change given our inability to reverse atmospheric warming.

Lake warming exhibits variability, with surface and bottom water temperatures showing diverse trends across lakes and within individual lakes at varying depths over time. Around the world, lake surface temperatures are increasing. A global study by O’Reilly et al. (2015) reported spatially varying increases in summer surface temperature, averaging at 0.34 °C/decade. However, the global trend is overlaid by variability due to regional or local conditions. This is exemplified by Bukaveckas et al. (2023) who reported variable temperature trends spanning from almost no increase at all to extreme values up to 0.7 °C/decade within 17 lakes of the Adirondack Mountain region, with an average increase (+ 0.27 °C/decade) close to the observed global trend reported by O’Reilly et al.

In contrast to surface temperatures, lake bottom temperatures are increasing less consistently and are even decreasing in many regions of the world (Bartosiewicz et al. 2019; Pilla et al. 2020; Bukaveckas et al. 2023). This phenomenon was also confirmed by various modelling studies (Kraemer et al. 2015; Schwefel et al. 2016; Shatwell et al. 2019). The resulting increasing difference between surface and bottom temperatures intensifies trends in lake stratification phenology. Shifts from polymictic or cold-monomictic to dimictic mixing regimes can be expected in the future. In dimictic or monomictic lakes, higher temperatures will result in extended periods of summer stratification and shortened periods with ice cover. However, the severity of these changes varies globally and depends on local lake characteristics (Woolway et al. 2021).

Besides increasing temperatures, changes in the mixing behaviour in seasonally stratified lakes affect the ecosystem indirectly by limiting the exchange of nutrients and dissolved oxygen (DO) between epilimnion and hypolimnion. However, the intensity of this secondary effect is still largely unknown. Hypolimnetic DO concentrations are decreasing in many lakes as a result of increased lake stratification (Foley et al. 2012; Sahoo et al. 2013; Schwefel et al. 2016; Jane et al. 2021). Additionally, reduced vertical transport is limiting the nutrient levels in the productive surface layer and can lead to the accumulation of large amounts of phosphorus in the deep hypolimnion of meromictic or oligomictic lakes (Yankova et al. 2017; Schwefel et al. 2019; Lau et al. 2020). However, local processes and anthropogenic impacts such as agricultural nutrient inputs or ongoing restoration measures often affect biogeochemical reactions in parallel to global warming. Therefore, contrary to mostly physically driven processes such as surface warming and changing lake mixing behaviour, predictions of ecological changes on a global scale are difficult to achieve. Studies need to consider local boundary conditions and their potential development to generate useful forecast scenarios which might help to set-up measures to mitigate potential negative climate effects and to make lake ecosystems more resilient against climate warming.

Enhancing ecosystem resilience is vital for adapting to climate change and should complement mitigation efforts (Howarth and Robinson 2024). The effects of global warming on lakes are documented to be similar to those resulting from eutrophication (Moss et al. 2011), thus any measures to reduce eutrophication can be considered as being climate-adaptive. For example, increased productivity arising from rising internal loading (North et al. 2014) and longer vegetation phases in a warmer climate could be compensated if nutrient loading from external sources is reduced. Therefore, limnologists call for adjustive measures targeting landscape features and nutrient mobilization to enhance the resilience (Meerhoff et al. 2022). While these statements are widely accepted by researchers, quantitative tools to demonstrate the resilience mediating impact of nutrient reduction are lacking. Hence, this study aims to address this gap by concentrating on hypolimnetic DO concentration, a highly sensitive indicator of lake water quality shown to be sensible to climate warming (Schwefel et al. 2016). To achieve this, firstly climate warming has to be quantitatively translated with respect to its consequences on temperature dynamics, stratification characteristics, and hypolimnetic DO. Subsequently, quantitative assessments of how improvements in nutrient loading or trophic state, respectively, would be able to improve hypolimnetic DO conditions in a warmer world, are necessary.

In this study, we investigate the impact of climate change on a series of German lakes. A large set of long-term monitoring data was used to calculate changes in surface and bottom temperatures, DO, total phosphorus (TP), and chlorophyll concentrations in 46 lakes with varying depth, trophic state and geographical position. 12 of these lakes were instrumented with sensor chains equipped with temperature and DO data loggers in high temporal and spatial resolution and allowed us to calibrate the numerical model FLake (Kirillin et al. 2011). The lake models were then run for the years 2005–2099 assuming different IPCC emission scenarios (IPCC 2014). The results of the numerical model were coupled with the DO model of Nkwalale et al. (2024) to obtain DO concentrations at the end of the stratified period. The goals of this study were (1) to identify trends in surface and bottom temperatures, their variability, and potential drivers; (2) to predict future changes in temperature and stratification phenology until the end of the twenty-first century; (3) to assess the impact of these changes on future hypolimnetic DO concentrations; and (4) to identify adaptive management reactions by eutrophication control and the corresponding impact from reducing primary productivity (e. g. in response to internal lake restoration measures or reducing external nutrient loading).

## Materials and methods

### Observational data

Lake temperatures in Germany were systematically measured since the end of the nineteenth century (e. g. Halbfass 1896; Thienemann 1928). While these early works rarely cover large periods of time, continuous long-term measurements started in the 1960s. Here we obtained 46 datasets covering at least 30 years (starting before or in 1990) using data from state agencies and the German environment agency (Table 1).

**Table 1:** Lakes used in the analysis of long-term monitoring data with measurements spanning over a period of more than 30 years. Lakes which were modelled by FLake are in bold font. Two lakes were numerically modelled but were excluded from the analysis of long-term monitoring data since their measurements did not cover the minimum period of 30 years. Data sources are listed in the last column. BE: Senatsverwaltung für Mobilität, Verkehr, Klimaschutz und Umwelt. Berlin (wasserportal.berlin.de). BY: Bayerisches Landesamt für Umwelt, Gewässerkundlicher Dienst Bayern (www.gkd.bayern.de/en/lakes). IGB: IGB Berlin (www.fred.igb-berlin.de). MV: Ministerium für Klimaschutz, Landwirtschaft, ländliche Räume und Umwelt Mecklenburg-Vorpommern. Abteilung 4—Wasser, Boden, Abfallwirtschaft, Immissionsschutz, Strahlenschutz, Fischerei, Referat 460—Gewässerkunde, Seenprogramm, Klimawandel. NS: Niedersächsischer Landesbetrieb für Wasserwirtschaft, Küsten- und Naturschutz, Seen-Kompetenzzentrum. UBA: Umweltbundesamt (German environment agency), Data according to state measurements

Lake name	Stratification s = stratified p = polymictic	Latitude (°)	Longitude (°)	Altitude (m)	Max. depth (m)	Area (km^2^)	Source
*Lakes used in the analysis of long-term monitoring data*							
Abtsdorfer See	s	47.91	12.91	420.2	20	0.84	BY
Ammersee	s	48.00	11.12	533	81.1	46.6	BY
Kleiner Arbersee	s	49.13	13.12	935	9.7	0.07	BY
**Arendsee (AS)**	**s**	**52.89**	**11.47**	**23.3**	**50**	**5.14**	**IGB**
Bannwaldsee	s	47.60	10.78	785.9	12	2.28	BY
Bodensee	s	47.63	9.37	395	251	536	UBA
**Breiter Luzin (BL)**	**s**	**53.36**	**13.47**	**84.3**	**58.3**	**3.45**	**IGB**
Chiemsee	s	47.86	12.40	518.2	73.4	79.9	BY
Dämeritzsee	p	52.42	13.73	32	4.5	0.93	BE
**Feldberger Haussee (HS)**	**s**	**53.34**	**13.45**	**84.3**	**12.5**	**1.31**	**IGB**
Großer Alpsee	s	47.57	10.18	724.6	22.7	2.47	BY
Hartsee	s	47.93	12.37	0.86	39.1	0.87	BY
Hopfensee	p	47.60	10.67	783.8	10.4	1.94	BY
Kochelsee	s	47.65	11.34	600	65.9	5.95	BY
**Laacher See (LS)**	**s**	**50.41**	**7.27**	**275**	**51**	**3.31**	**UBA/IGB**
Langbürgner See	s	47.90	12.36	530	37.3	1.04	BY
Lieps	p	53.45	13.16	14.8	3.8	4.31	IGB
**Müggelsee (MS)**	**p**	**52.43**	**13.65**	**32.3**	**7.7**	**7.4**	**IGB**
Niedersonthofener See	s	47.63	10.26	703.3	21.3	1.35	BY
Pelhamer See	s	47.93	12.35	530	21.3	0.72	BY
Pilsensee	s	48.03	11.19	534	17.1	1.95	BY
Plauer See	s	53.47	12.31	62	25.2	38.4	MV
Rachelsee	s	48.98	13.40	1070	13.5	5.7	BY
Riegsee	s	47.70	11.23	658.6	15.4	1.97	BY
**Schaalsee (SA) (main basin)**	**s**	**53.61**	**10.93**	**34.8**	**72.3**	**23.5**	**MV**
Schaalsee (Zarrentiner Becken)	s	53.56	10.93	34.8	63.4		MV
**Schmaler Luzin (SL) (main basin)**	**s**	**53.32**	**13.44**	**84.3**	**33.5**	**1.45**	**IGB**
Schmaler Luzin (Carwitzer Becken)	s	53.31	13.43	84.3	33		MV
Schwerinersee (Außensee)	s	53.68	11.46	37.8	52.4	61.54	UBA/MV
Seddinsee	p	52.39	13.68	35	7	2.69	BE
Simssee	s	47.87	12.24	470	22.5	6.49	BY
Staffelsee (Nord)	s	47.69	11.16	648.6	39.4	7.66	BY
Starnberger See	s	47.92	11.32	596	127.8	58.36	BY
Stechlinsee	s	53.15	13.03	59.6	68	4.12	IGB
Steinberger See	s	49.28	12.16	364	50	1.84	BY
Steinhuder Meer	p	52.47	9.33	38	2.9	29.1	NS
Tegernsee	s	47.73	11.73	725.5	72.6	8934	BY
**Tegeler See (TS)**	**s**	**52.58**	**13.25**	**31.4**	**15.9**	**4539**	**BE/IGB**
**Tiefwarensee (TW)**	**s**	**53.53**	**12.69**	**63**	**23.6**	**1.41**	**IGB**
**Tollensesee (TO)**	**s**	**53.51**	**13.21**	**14.8**	**31.3**	**17.9**	**IGB**
Waginger See	s	47.94	12.78	442.1	27	6.61	BY
Walchensee	s	47.59	11.35	800.8	189.5	16.27	BY
Wannsee	p	52.43	13.17	32	9.8	2.73	BE
Weißensee	s	47.57	10.64	787.3	24.7	1.35	BY
Wörthsee	s	48.06	11.18	562	34	4.32	BY
Zwischenahner Meer	p	53.20	8.02	5	6	5.5	NS
*Modelled, but less than 30 years of long-term monitoring*							
**Großer Plöner See (PS)**	**s**	**54.13**	**10.41**	**21**	**56.2**	**28.4**	**IGB**
**Titisee (TT)**	**s**	**47.89**	**8.15**	**845.6**	**39**	**1.07**	**IGB**

**Fig. 1:**
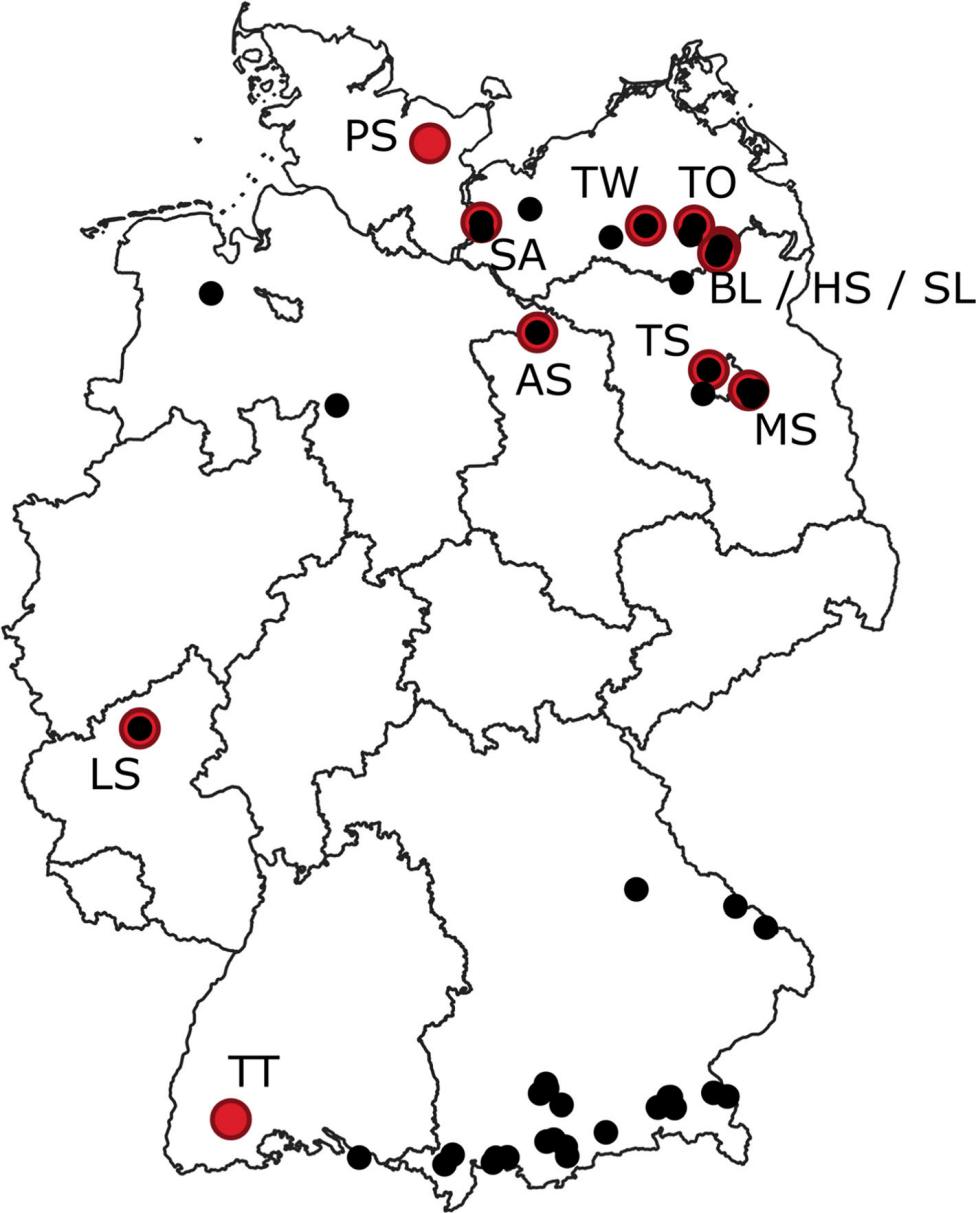
Lakes modelled with FLake (red) and lakes included in the long-term monitoring study (black) within the administrational boarders of Germany. For the abbreviations see Table 1

Maximum lake depths span from 3 m in the shallow, polymictic Steinhuder Meer to 251 m in Lake Constance, whilst lake areas are from 11 ha (Kleiner Arbersee) to 536 km^2^ (Lake Constance). All major geographical regions (Prealps, Central Uplands and North German Plain) are covered, with most lakes situated in the Prealps (*n* = 22) and the North German Plain (*n* = 20), and only four lakes in the midland region due to the overall lower abundance of large natural lakes there. Some multi-basin lakes were monitored in more than one side basin which were then counted separately. Eight lakes were polymictic while the remaining datasets represent dimictic or warm-monomictic lakes. The locations of the lakes are shown in Fig. 1 and a complete list is given in Table 1. In total 1403 lake-years with at least one temperature and DO measurement were available of which 968 contained additional TP data and 211 Chlorophyll-a (Chl-a) data. The temporal resolution was generally at least six measurements/year after 1990, but often only sporadic beforehand. Therefore, the period before 1990 was not analysed quantitatively.

We calculated seasonal trends of surface and bottom temperatures, bottom DO concentrations, and correlations between temperature, TP, Chl-a and DO. Surface (uppermost 1 m) and bottom (deeper than 80% of the maximum depth; only calculated for dimictic lakes) data were calculated as seasonal averages. Seasonal averages (with spring defined as March, April, and May, Summer as June, July, and August, fall as September, October, and November and winter as December as well as February and March of the following year) were calculated for all years in which at least one measurement was performed during the corresponding season. Annual rates of summer DO depletion at the bottom were calculated as linear fits over periods in which bottom layer DO concentrations were decreasing but not anoxic yet (see examples in Figure S1). Annual average temperatures were calculated if at least six measurements were performed in 1 year. Because sampling dates of individual lake monitoring programs were inconsistent, these annual trends are not a priori comparable between lakes.

Since state agencies are legally required to take samples during the vegetation period and are not always continuing their monitoring program during winter, trends could also be biased due to an overrepresentation of summer data in the data set. To assess the former problem, we calculated an average measurement day as arithmetic mean of the calendar days of all measurements in a particular year. The average standard deviation of this quantity was approximately 17 days. In 9 out of 45 lakes, we found a significant correlation between the average measurement day and the measured average surface temperature. However, only in one of these lakes (Staffelsee) a significant trend in the average measurement date was observed. This was caused by a higher number of measurements during late summer and autumn in the later period of the time series (2010–2020). To correct for this bias, measurements in October, which typically only happened in the second half of the time series, were removed for Staffelsee. The problem of an overrepresentation of summer date was addressed by comparing the measured annual trends with the average of the four seasonal trends if both data were available. Here we found a significant correlation (*r* = 0.67, *p* << 0.01) and almost no bias (0.01 °C/Decade or ~ 2% of the measured trend).

In addition to these low-frequency monitoring data, sensor chains equipped with DO and temperature loggers were deployed in a set of German lakes (Table 1; Fig. 1). Measurements frequency was at least hourly in at least six depths for temperature (at least three to four depths for DO). The sensor chains were typically deployed close to the deepest point of the lake, vertical resolution varied depending on the lake and was usually higher in the epilimnion. Details about the sensor chains can be found in Hupfer et al. (2022).

Using the temperature chain data, stratification was determined based on the difference between surface and bottom temperatures. Different definitions of lake stratification exist (Engelhardt and Kirillin 2014). Here, we defined a lake as stratified when the surface temperature was at least 2 °C warmer than the bottom temperature. A temperature difference of 2 °C corresponds to a density difference of ~ 0.07 kg/m^3^ for typical temperatures during complete mixing in fall or early winter. Temperatures were linearly interpolated to 1 m resolution and the average between 1 and 4 m depth was defined as surface temperature. As the first temperature logger was typically located 1 m below the surface, we decided to ignore the first meter to avoid uncertainties concerning the interpolation to the surface. As bottom temperature, we defined the temperature 1 m above the sediment.

### Numerical modelling

In total 12 lakes with high-frequency temperature data for calibration were modelled using the one-dimensional model FLake. FLake simulates the thermal structure of a lake based on the concept of self-similarity using a two-layer approach with an upper mixed layer separated from the bottom layer (Mironov 2008; Kirillin et al. 2011). It predicts the mixed-layer depth, and the temperature of both layers with high numerical efficiency and was already used in various studies dealing with the impact of climate change on lakes (Golosov et al. 2012; Shatwell et al. 2019; Maberly et al. 2020; Woolway et al. 2020).

Air temperature (°C), solar radiation (W/m^2^) wind speed (m/s), water vapour pressure (mbar), and cloud coverage (–) are required as meteorological forcing for Flake. Additional input parameters are lake depth, average extinction coefficient and wind fetch. Lake depth was assumed to be constant throughout all model results including the calibration and validation as well as the predictions based on IPCC emission scenarios. For calibration, models were run for the years 2012–2020 using meteorological forcing from the nearest meteorological station. For Lake Titisee, meteorological data close to the shore were only available for 2019–2021, hence the calibration was done during this time. The distance from the nearest station varied between few hundred metres from the shore (Titisee) to 40 km in the case of Lake Müggelsee. Results were then compared to measurement data. One selected year of the data was used for calibration and the remaining data (if existent) for validation (Table S1). In the unstratified Lake Müggelsee, we only considered surface waters for calibration and validation. Since measurement chains were only deployed for short periods in some of the lakes, validation could not be performed everywhere.

In FLake, the temperature is modelled assuming a simplified two-layer approach (Mironov 2008). Although Mironov et al. (2010) report that the mean depth is the best choice, our calibration obtained the best results with lake depths being close or slightly lower than the lake mean depth in deep lakes (maximum depth > 30 m) and between mean and maximum depth for shallow lakes. Although no field data for annually averaged light extinction exist, extinction coefficients found by calibration were close to values expected from sporadic measurements of Secchi disc depth (*z*_SD_) assuming the extinction coefficient *ε* to be *ε* = *k* *z*_SD_^−1^. Data from Lake Arendsee suggest *k* = 1.4 to be the best conversion factor which is in line with findings from Fink et al. (2014) and Schwefel et al. (2016) for large perialpine lakes. Best results were often found with wind fetches smaller than the average lake area, probably because of a systematic bias with wind speeds measured at meteorological stations being in average higher than wind speeds directly above the lake.

Model results for the surface were acceptable for a simplified one-dimensional model and root mean square errors (RMSE) were between 0.7 and 2.1 °C with correlations between measured and modelled temperatures being always higher than 0.95. Nash–Sutcliffe-Efficiencies (NSE) were between 0.90 and 0.99 (Table S1). The model often failed to reproduce bottom temperatures correctly given the simplified two-layer approach for the temperature profile and the neglection of lateral transport processes. RMSE were similar to values found at the surface, but the model often failed to reproduce the gradual warming during summer stratification correctly. On the other hand, average winter bottom temperatures were often overestimated. The average summer temperature—a key variable for the oxygen model (see below)—was generally estimated more precisely. Here the bias during validation was generally less than 0.6 °C. The correlation between measured and modelled temperature was significantly lower than at the surface (between 0.30 and 0.84). Particularly at the bottom of the deep lakes, temperatures remained very stable (the standard deviation of the measured temperature was between 0.1 and 0.4 °C in the deep lakes). As NSE compares model performance to the performance of the mean value as predictor, the poor model performance therefore resulted in negative NSE in many cases (Table S1). However, since average summer temperatures and stratification duration were reproduced well, the model provides reliable input data to estimate oxygen concentrations at the bottom (see below).

The accuracy of stratification duration was assessed based on in total 36 years with data for stratification onset or end. Here, the beginning of the stratified period was predicted well with a bias of 0.4 days and a RMSE of 6.1 days. The end of stratification was in average 7.5 days shorter in the simulations compared to the observations, the RMSE was 16.8 days. As a result, the stratification duration was slightly underestimated in the model (bias of − 7.7 days, RMSE = 16.5). Modelled and observed stratification duration as well as beginning and end of the stratified period correlated significantly (*r* = 0.72, 0.78, and 0.68, respectively; *p* << 0.1 in all cases).

To predict changes in the thermal structure of lakes under future climate conditions, the calibrated models were then forced by a series of climate models assuming future atmospheric CO_2_-concentrations according to the IPCC scenarios RCP 2.6, RCP 4.5, and RCP 8.5. Here we used climate model results provided by the German meteorological service. They provide a set of 16 combinations of global circulation models and regional downscaling models (GCM/RCM) (“*Kernensemble*”, see Table S2). The *Kernensemble* is designed to keep the full variability in possible climate developments predicted by in total 45 GCM/RCM-combinations (“*Referenzensemble*”) but reduces redundancy by ignoring model outputs with very similar results. The spatial resolution of the climate model outputs is 5 km and lake models were run using averaged values from the grid point in which the lake is located and the eight neighbouring grid points. Comparisons between the measurements at the closest meteorological station and the model inputs showed no systematic differences. The model predictions were mostly within one standard deviation of the measurements (see example in Figure S2). The *Kernensemble* covers the period 2006–2099, its historical reference period from 1970 to 2005 was additionally run.

Numerous numerical models with varying approaches and different levels of complexity were developed to reproduce physico-chemical and biological properties, focusing either on single variables (e. g. hypolimnetic oxygen concentrations; Deyle et al. 2022) or simulating different trophic stages by reproducing the whole nutrient and DO budget and different groups of phyto- and zooplankton (e. g. Rucinski et al. 2014; Krishna et al. 2022). As more complex models depend on a large amount of state variables, they need careful calibration. For a prediction on larger scales over long periods of time with limited observational data for calibration, simple statistical approaches are often a more promising approach.

Here we decided to apply the hypolimnetic DO depletion model described in Nkwalale et al. (2024) to reproduce the DO concentrations as one key quantity for the lake ecosystem. The model is based on typical hypolimnetic DO depletion rates for eutrophic, mesotrophic and oligotrophic lakes derived from statistical analysis of a large set of DO depletion rates reported in the literature. The model predicts DO concentrations in the hypolimnion during the stratified summer period as function of trophic state (as defined in Carlson 1977), temperature, stratification duration, and the initial DO concentration after spring mixing. Assuming 100% saturation at the onset of stratification, the DO concentration at the end of the stratified period each year could then be calculated using bottom temperatures and stratification durations obtained from the numerical model FLake (see above). A validation based on several German lakes gave a root mean square error of 1.5 mg/L and a correlation coefficient of 0.6, signifying a good model performance given the simplicity of the approach (Nkwalale et al. 2024). The Matlab-function used to run the model is given in the supplementary information (Text S1). All post-processing was done using Matlab R2023b (https://matlab.mathworks.com).

For the prediction of DO concentrations at the end of the century, two different sources of socioeconomic uncertainties must be considered: (1) on a global level the development of CO_2_-concentrations in the atmosphere which are covered by the IPCC emission scenarios, and (2) changes in the trophic state caused by altering nutrient inputs from the catchment which depend on local decisions. To take the latter uncertainty into account and to assess the importance of local management measures relative to global effects of climate change, we considered two possible developments. Scenario A assumes the trophic state to be constant during the whole simulation period. In Scenario B, we assume that the trophic level improves in the second half of the twenty-first century. Consequently, nowadays eutrophic lakes become mesotrophic, mesotrophic lakes become oligotrophic, but conditions do not change in lakes which are already oligotrophic today. Scenario B reflects possible measures to limit nutrient inputs, e. g. by changing land-use in the catchment area and can be considered as proactive management response to enhance resilience against climate warming. Scenario B can also be used to assess the effectiveness of ongoing measures to reduce nutrient loads in light of the parallel impact of climate change.

## Results

### Long-term monitoring data

All lakes showed typical mixing behaviour for a temperate region. Eight of the lakes were shallow and polymictic while the remaining 38 lakes mixed one or two times per year with a long stratification period during summer and, for the dimictic lakes, a shorter one in winter. Water surface temperatures were strongly correlated with regional air temperatures (*r* = 0.80, *p* < 0.001). In contrast, the correlation between water and air temperatures was much weaker at the bottom of stratified lakes (*r* = 0.28, *p* < 0.001). Instead, bottom temperatures also depended on lake depth and surface area with deeper and smaller lakes experiencing lower bottom temperatures. A linear model with air temperature, lake depth, and lake area as independent variables described the bottom temperatures fairly well (*r*^2^ = 0.40, *p* < 0.001; Table S3). Adding interaction terms improved the model only marginally (*r*^2^ = 0.42, *p* < 0.001). These findings indicate that surface temperatures mostly directly follow their meteorological forcing while bottom temperatures depend primarily on lake morphometry.

**Fig. 2:**
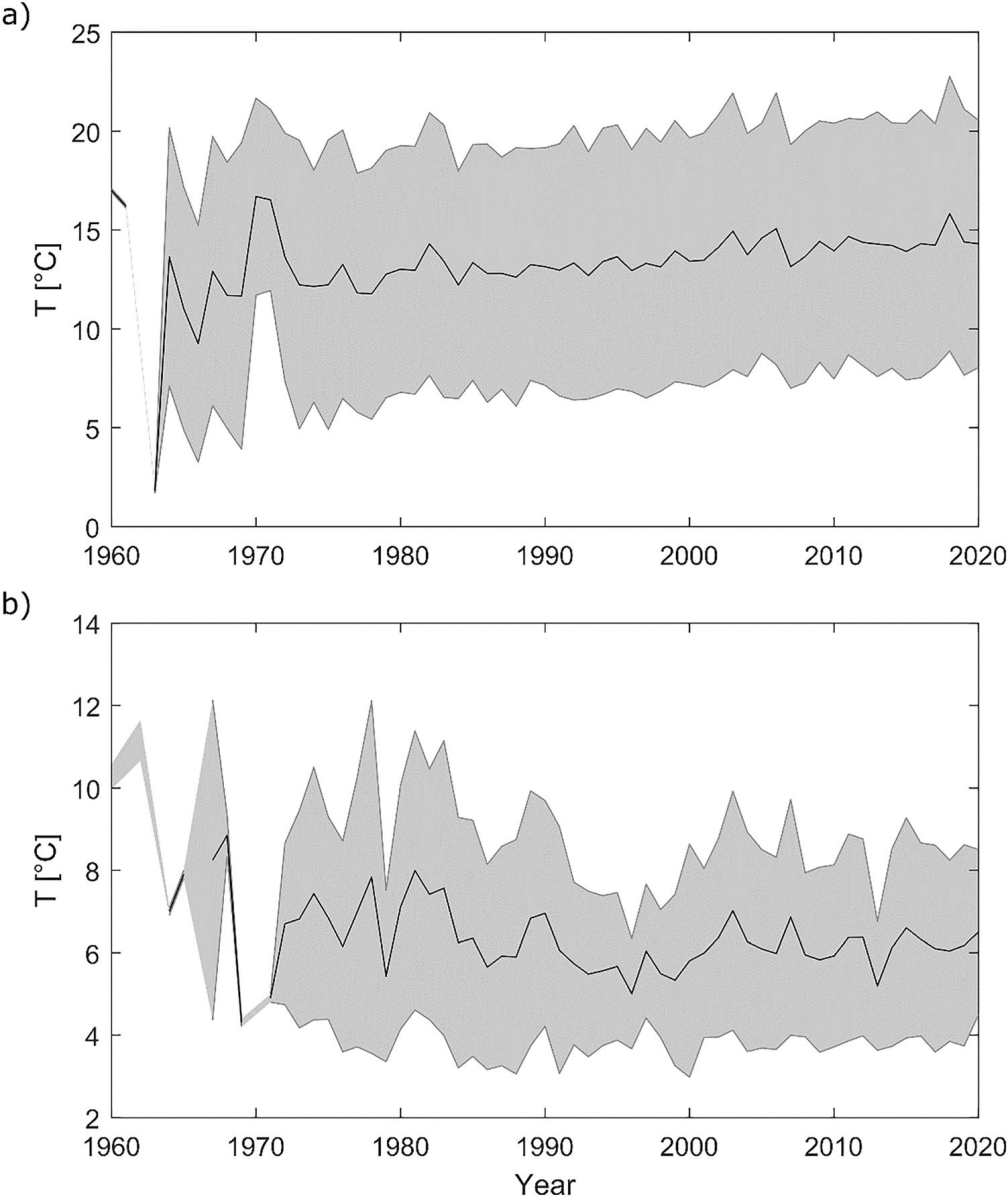
Annual surface (**a**) and bottom (**b**) temperatures averaged over all 46 lakes, only a very small number of lakes had data ranging back before 1980, hence the results for the early period are not representative for the entire dataset. The solid lines give the mean values, the shaded area the standard deviation

**Fig. 3:**
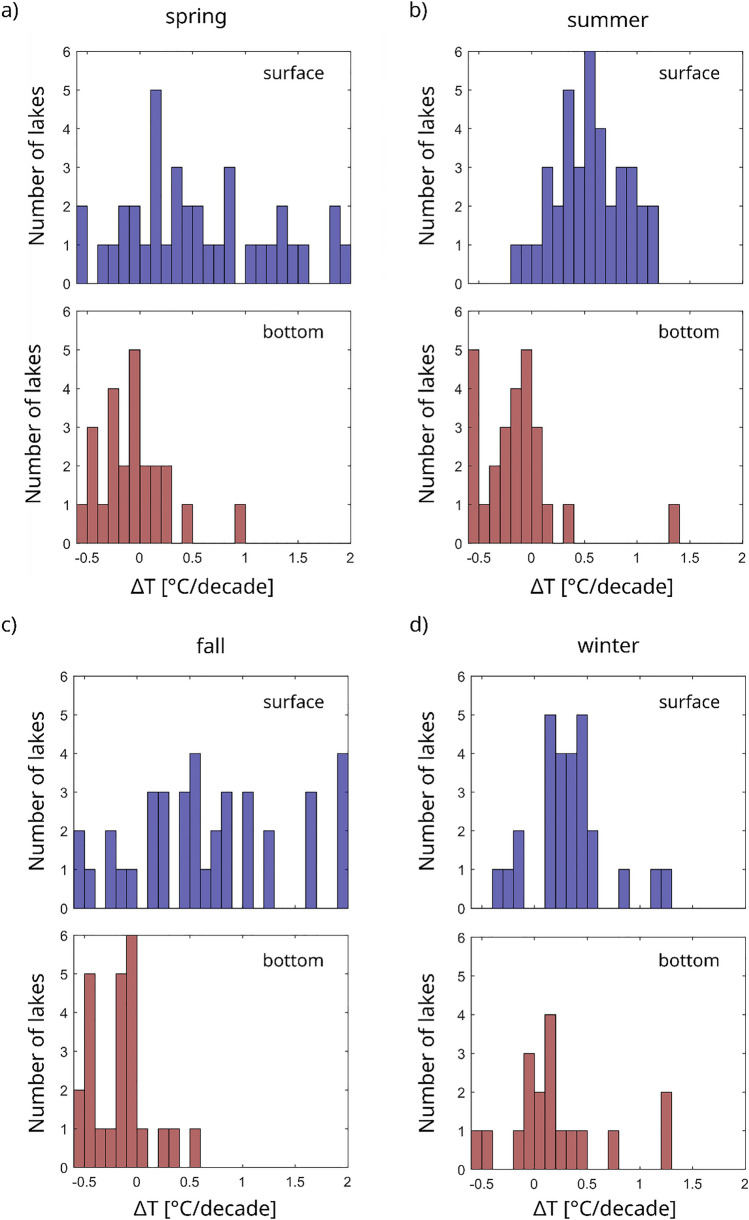
Histograms of average gradients in surface and bottom temperatures between 1990 and 2020 in spring (**a**), summer (**b**), fall (**c**), and winter (**d**). Bottom temperatures of polymictic lakes were excluded

Surface temperatures were increasing by + 0.5 ± 0.1 °C/decade in average between 1990 and 2020 (Fig. 2a). In parallel, bottom temperatures in stratified lakes decreased slightly, changing by − 0.1 ± 0.1 °C/decade (Fig. 2b), resulting in a stronger gradient between bottom and surface temperatures. While changes in surface temperatures were highest in fall (+ 0.6 ± 0.2 °C/decade) and lowest in winter (+ 0.3 ± 0.1 °C/decade), bottom temperatures increased in winter (+ 0.2 ± 0.1 °C/decade) but decreased in all other seasons between 0.1 and 0.2 ± 0.1 °C/decade (Fig. 3). Trends in surface or bottom temperatures were not correlated to mixing behaviour, mean depth, or geographical features such as altitude. Surface temperatures increased more strongly in lakes situated in the northern lowlands (+ 0.7 °C, standard deviation 0.4 °C/decade) compared to alpine or perialpine regions (+ 0.5 °C, standard deviation 0.5 °C/decade). However, this difference was not statistically significant (*p* = 0.3).

Bottom DO concentrations were typically close to saturation in winter and spring and decreased during the stratified period in summer. Winter hypoxia at the lake bottom occurred rarely. Concentrations below 2 mg L^−1^ were only found in 15% of all winter measurements. In contrast, 51% of the summer measurements and even 62% of the fall measurements fell below this threshold. The occurrence of hypoxic conditions was increasing since 1980 in parallel to the air temperatures especially in fall (Figure S3). Bottom DO concentrations during summer were correlated with lake depth and anticorrelated with TP concentrations and temperature at the bottom. They could be described with a multiple linear model (*r*^2^ = 0.39; Table S4). The inclusion of interaction effects did not improve the model result significantly (*r*^2^ = 0.40). Summer rates of DO depletion at the bottom were 68 ± 39 mg m^−3^ d^−1^. Since DO depletion rates increase with depth (Schwefel et al. 2018; LaBrie et al. 2023), average rates for the whole hypolimnion are lower but were not calculated due to a lack of bathymetric data of many lakes and difficulties with vertical interpolation of often limited DO measurements. Trends in bottom DO depletion were highly variable with exactly 50% of the lakes (19 out of 38) showing decreasing rates of DO depletion and the other half experiencing increasing rates between 1990 and 2020.

Like the DO concentrations, trends in TP depended strongly on local conditions. TP concentrations were tendentially decreasing (on average over all lakes by ~ 0.6 mg m^−3^ year^−1^ at the surface but only 0.1 mg m^−3^ year^−1^ at the bottom), most likely reflecting the impact of lake management measures. Chl-a concentrations tended to decrease in parallel. However, trends in Chl-a concentrations averaged over all lakes were not statistically significant, neither annually averaged nor for individual seasons (*p* > 0.2 in all cases). Overall, Chl-a was strongly correlated with TP concentrations (*r* = 0.52; *p* < 0.001), but only weakly correlated with temperature if data from all seasons were considered (*r* = 0.07; *p* < 0.001). Chl-a was positively correlated with temperature during spring (*r* = 0.09; *p* = 0.001), but negatively correlated with temperature during summer (*r* = − 0.13; *p* < 0.001) and (not significantly) in fall (*r* = − 0.05; *p* = 0.09). During summer and fall, temperatures were also negatively correlated with TP (*r* = − 0.07 and *r* = − 0.11; *p* < 0.001 in both cases).

### Numerical simulations

#### Changes in lake temperature and stratification until the end of the century

**Fig. 4:**
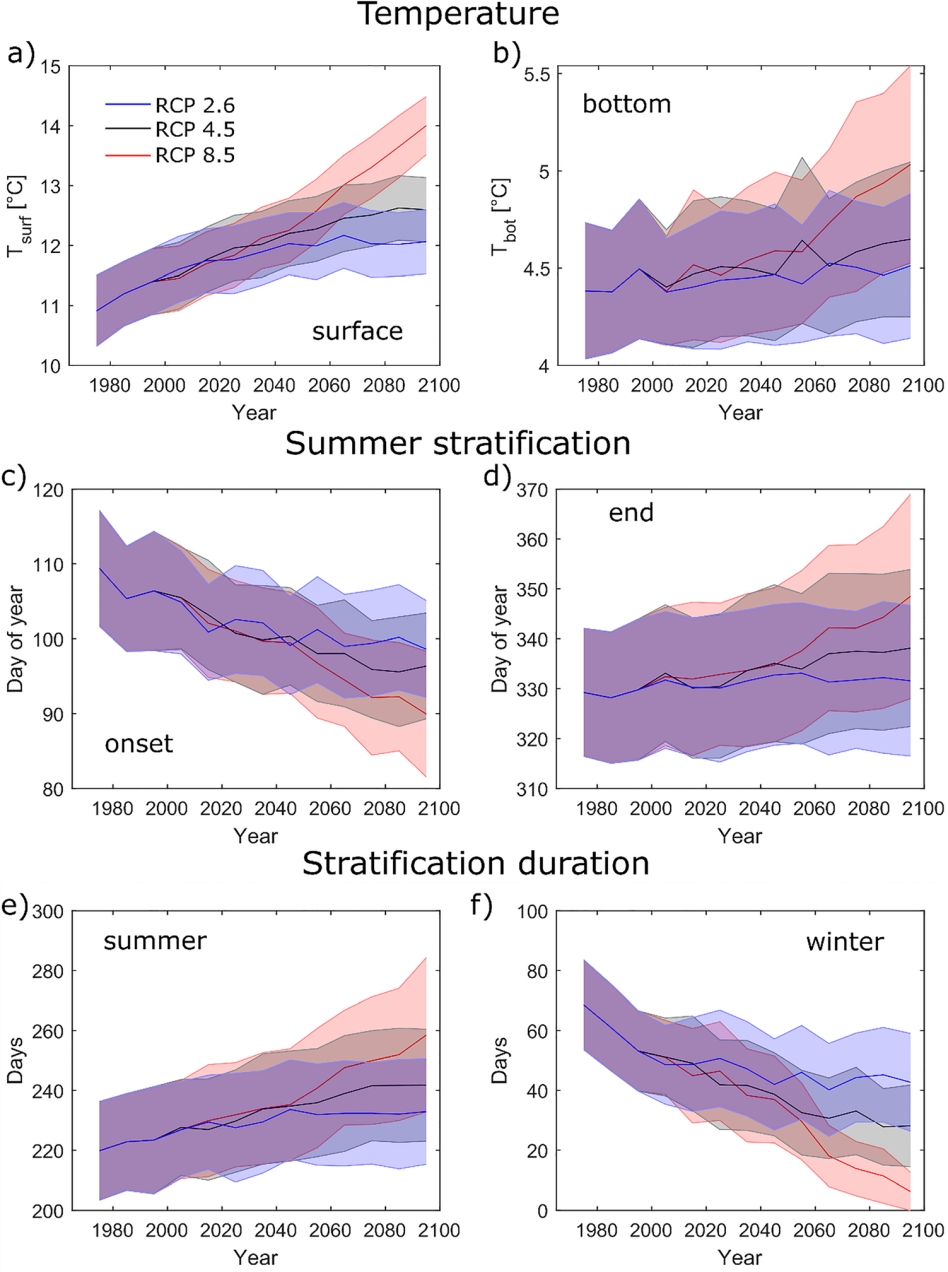
Mean surface temperatures (panel **a**), bottom temperatures (**b**), onset (**c**), end (**d**), and duration of the stratified period during summer (**e**) and duration of the stratified period during winter (**f**) for the emission scenarios RCP 2.6 (blue), RCP 4.5 (black) and RCP 8.5 (red). The shaded areas give the standard derivation, the lines the mean of all modelled lakes except for the polymictic Lake Müggelsee

Lake surface temperatures were consistently higher towards the end of the simulation period compared to the first decade of simulations 2006–2016. Until the middle of the century, surface temperatures developed similarly in all emission scenarios. However, considerable differences were observed in the second half of the century (Fig. 4a). In RCP 2.6, temperatures reached a maximum in the decade 2060–2070 and decreased afterwards slowly. In RCP 4.5, the increase in surface temperatures slowed down but did not reach a maximum before approximately 2090. In contrast, warming accelerated in RCP 8.5 in the second half of the century with temperatures continuously increasing at even higher rates compared to reference period. The average rate of temperature increase between 2006 and 2099 was 0.3 °C/decade in scenario RCP 8.5, 0.18 °C/decade in Scenario RCP 4.5, and only 0.04 °C/decade in Scenario RCP 2.6. In contrast to the surface temperatures, bottom temperatures did not change significantly in RCP 2.6, and increased at considerably slower rates compared to surface temperatures in RCP 4.5 (+ 0.02 °C/decade) and RCP 8.5 (+ 0.05 °C/decade).

Both, increasing temperature gradients between surface and bottom waters and higher average water temperatures lead to a stronger stratification during summer. Averaged over all stratified lakes, the stratification duration increased by 0.5 days/decade in RCP 2.6, 1.9 days/decade in RCP 4.5, and 3.7 days/decade in RCP 8.5. Despite considerable uncertainties in predicting the exact timing of the onset and end of stratification (as discussed in the “Methods” section), the modelled and observed stratification duration correlated well during calibration. Consequently, we believe that the predicted long-term trends in stratification until the end of the century give a robust estimate of future developments. Until the end of the century, the duration of summer stratification extended by 38 days on average compared to the decade 2006–2016 for RCP 8.5, by 22 days for RCP 4.5, and by 13 days for RCP 2.6. During the last decade of the simulation, the stratification lasted 19, 9, or 2 days longer depending on the emission scenario and started 19, 14, or 10 days earlier. Stratification end changed more strongly during the end of the simulation period for RCP 4.5 and especially for RCP 8.5. By contrast, stratification onset shifted to earlier dates at an approximately constant rate in RCP 8.5, and the shift was decelerating during the second half of the simulation period for the two other emission scenarios (Fig. 4c, d). While the changes in stratification onset were similar in all lakes, the end of stratification changed more strongly in the deep lakes (Figure S4); Table 1).

Inverse stratification during winter decreased significantly. The average time during which surface temperatures were at least 0.2 °C below bottom temperatures is depicted in Fig. 4e. In the last decade of scenario RCP 8.5, the deep lakes Arendsee, Laacher See, Plöner See, and Schmaler Luzin did not stratify at all during winter, indicating a shift from dimictic to monomictic mixing regimes. This was not observed in the other scenarios RCP 2.6 and 4.5. Similarly, the only investigated polymictic lake Müggelsee showed no sign of transition to a dimictic state although there were longer periods of intermittent stratification during summer in RCP 4.5 and RCP 8.5.

#### Oxygen model

**Fig. 5:**
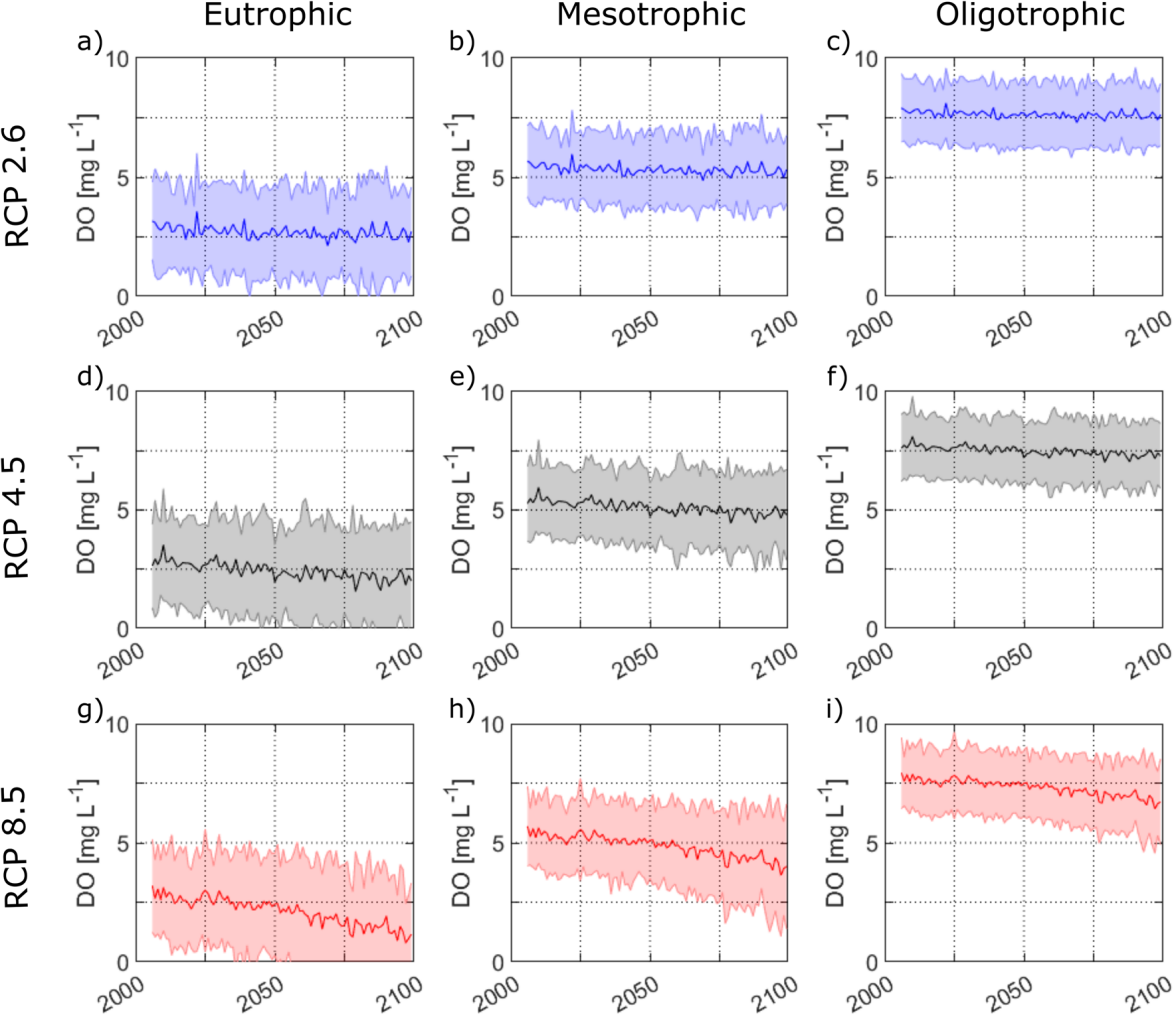
Predicted oxygen concentrations at the end of summer in the modelled lakes assuming eutrophic (**a**, **d**, **g**), mesotrophic (**b**, **e**, **h**) or oligotrophic (**c**, **f**, **i**) conditions in all eleven modelled stratified lakes until 2099 according to the IPCC emission scenarios RCP 2.6 (**a**–**c**), RCP 4.5 (**d**–**f**) and RCP 8.5 (**g**–**i**). The solid line represents the mean value, the shaded area the range of all predicted oxygen concentrations

**Fig. 6:**
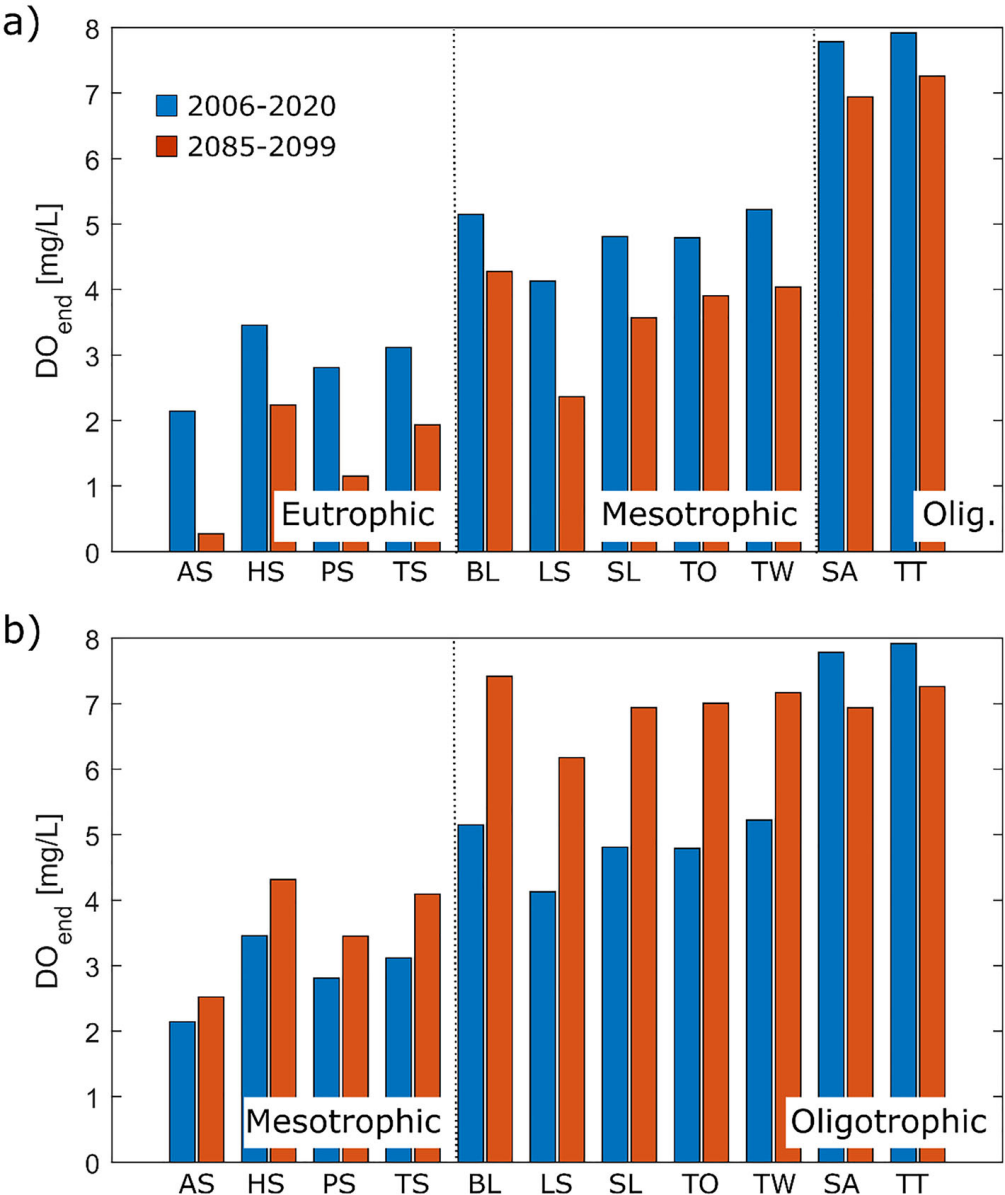
Predicted oxygen concentrations at the end of the stratified summer period during the beginning and the end of the simulation period assuming the emission scenario RCP 8.5 in the 11 simulated lakes with summer stratification (for abbreviations, see Table 1). Trophic state is assumed to be constant in panel a. In panel b, the trophic state is assumed to improve by one level in mesotrophic and eutrophic lakes in the second half of the twenty-first century

The predicted change in DO concentrations assuming (hypothetical) eutrophic, mesotrophic or oligotrophic conditions is depicted in Fig. 5. In Fig. 6, the predicted average DO concentration at the end of the stratified period during the first 15 years and the last 15 years of the simulation period is shown for both simulated scenarios. In Scenario A (constant trophic state as currently observed; Fig. 6a), DO concentrations were consistently lower at the end of the century compared to the beginning of the simulation period. Average concentrations at the end of summer decreased from 4.9 mg L^−1^ on average in the first 15 years of simulation to 3.5 mg L^−1^ for RCP 8.5, 4.2 mg L^−1^ for RCP 4.5, and 4.6 mg L^−1^ for RCP 2.6 in the last 15 years (2085–2099). Changes were largest in deep lakes where the end of stratification period changed most. In RCP 8.5, the decrease corresponds to an additional DO loss during the stratified period by up to 50 g O_2_ per m^2^ of lake area in the deep lakes Arendsee and Laacher See. The losses were lower (7–20 g O_2_ per m^2^ of lake area) in the other lakes with lower mean depth and/or lower productivity. As bottom temperatures increased much less than stratification duration, DO depletion rates stayed almost constant, and the extension of the stratification period was the main driver for changes in the DO content. A completely different situation evolves in scenario B (improving trophic state; Fig. 6b). While the impact of climate change can still be seen in the continuously oligotrophic lakes Titisee and Schaalsee, end-of-summer DO concentrations increase in all other lakes resulting in an average increase of DO concentrations at the end of summer by 1.8 mg L^−1^ in RCP 2.6, 1.5 mg L^−1^ in RCP 4.5, and 1.1 mg L^−1^ in RCP 8.5.

## Discussion

### Trends observed in the monitoring data

The observed increase in surface temperatures of 0.5 °C/decade between 1990 and 2020 exceeds the trend in air temperatures (+ 0.43 °C/decade on average in Germany between 1990 and 2020). It also exceeds the global trend in lake temperatures of + 0.24 °C/decade derived from a combination of satellite data and numerical modelling (Tong et al. 2023). During summer, temperature trends (+ 0.7 °C/decade) were also higher than the globally observed increase by + 0.34 °C/decade between 1985 and 2009 (O’Reilly et al. 2015). In O’Reilly et al. (2015) as well as in Tong et al. (2023), highest warming was observed in temperate and arctic regions. Their dataset contained four German lakes with an average summer surface temperature increase of + 0.55 °C/decade which confirms that summer surface temperatures in Germany increase faster than the global average. Very similar temperature increases (+ 0.58 °C/decade in average) were observed for 10 European lakes by Dokulil et al. (2021) but trends were higher than the change reported by Piccolroaz et al. (2020) for temperate regions worldwide. Albeit not statistically significant, our observation that alpine and perialpine lakes warm less quick than lakes in Northern Germany is in line with the observed patterns in O’Reilly et al. (2015). In general, surface lake warming was highly variable, which could be partially caused by low measurement resolutions, and no statistically significant relationship between trends in temperature and morphometrical or geographic characteristics could be found.

Unfortunately, it is not possible to estimate the duration of stratification reasonably with data in approximately monthly resolution. However, the increasing difference between surface and bottom temperatures during summer indicates a consistently higher temperature gradient indicating stronger stratification. This, in turn, hints to a delayed timing of winter mixing. The decreasing bottom temperature in summer and fall is indicative for an earlier and probably also more abrupt onset of stratification since bottom temperatures are depending on the duration and the temperatures during spring mixing. Since there are no increasing trends in nutrient concentrations or Chl-a visible in our data, the decreasing bottom DO concentrations in summer and fall can be interpreted as a result of a longer stratification period. Although no complete regime shift from monomixis to meromixis was observed in our study, longer stratification also implies a delayed replenishment of nutrients into the epilimnion from the aphotic hypolimnion. The anticorrelation between summer and autumn temperature and Chl-a and TP concentrations indicate that increased stratification already affects the nutrient budget and primary production. However, it is difficult to quantify these effects based on limited data in typically monthly resolution. Further studies using high-frequency monitoring data and more complex numerical models would be appropriate tools to improve the understanding of the impact of delayed fall mixing on primary production.

Hypoxia was widespread in lake hypolimnia during summer. It is caused by limited transport of DO through the thermocline in combination with ongoing DO depletion processes in the hypolimnion. While the former is controlled by physics, the latter are biological processes dependent on the availability of degradable organic material and are largely controlled by TP concentrations, lake depth, and hypolimnetic temperature as already established by Cornett and Rigler (1980) and confirmed by our dataset. Since deepwater temperatures showed no significant trend, climate change is likely to affect DO concentrations mainly indirectly by an extension of the stratified period, but not by a direct increase of rates of DO depletion. Therefore, DO concentrations can be controlled by nutrient control. Remedial measures are extremely costly (i.e., artificial aeration), hence lowering depletion rates by nutrient control remains the most effective way to reduce the occurrence of hypoxic conditions in lake hypolimnia.

### Predicted changes in temperature and stratification

Climate projections depend not only on the assumed greenhouse gas emissions but also vary depending on the chosen combination of global circulation models and regional downscaling models. Since the *DWD-Kernensemble* is designed to cover the whole spectrum of possible climate projections based on a large variety of GCM/RDC combinations, the standard deviation of annual mean air temperatures between different GCM/RDC-combinations was high (between 0.7 and 0.9 °C depending on the emission scenario). Future trends in the development of annually averaged surface water temperatures spanned between 0.18 and 0.38 °C/decade for RCP 8.5, between 0.05 and 0.21 for RCP 4.5, and 0.03 and 0.05 for RCP 2.6 depending on the chosen GCM/RDC-combination. A similar variability was observed by a global study of Golub et al (2022) who report between 2.4 and 5.3 °C Temperature change until the end of the century for RCP 8.5 depending on the chosen GCM. This exceeds the variability between different models observed while using an ensemble modelling approach with various one-dimensional lake models but identical meteorological forcing (e. g. Perroud et al. 2009; Feldbauer et al. 2022; Golub et al. 2022). Therefore, it can be concluded that the main uncertainty when predicting future lake surface temperatures is the development of the meteorological conditions although this is not necessarily true for other parameters (Wynne et al. 2023).

Our model results predict a lower trend in surface water temperatures compared to air temperatures according to the *DWD-Kernensemble* which is in line with the predictions by Golub et al. 2022 and observational findings (Tong et al. 2023)*.* Averaged over all climate models included in the *DWD-Kernensemble*, annually averaged air temperature changes by + 0.38 °C/decade on average for RCP 8.5 while annually averaged water temperatures increase only by + 0.30 °C/decade in our study. Lower trends in water temperatures compared to air temperatures were also observed in scenarios RCP 4.5 and 2.6 (0.11 and 0.04 °C/decade, respectively, for water as opposed to 0.18 and 0.06 °C/decade for air). Similarly, lower increases in water temperatures in comparison to air temperatures were found in other modelling studies using different GCM/RDC-combinations and lake models (Råman Vinnå et al. 2021; Golub et al. 2022). Tong et al. (2023) ascribe this phenomenon to increased water evaporation under warmer air temperatures. The trends in water temperatures for RCP 4.5 were comparable to those reported by Shatwell et al. (2019) for a set of four lakes in northern Germany (0.13 °C/decade on average). Råman Vinnå et al. (2021) predict a higher increase of + 0.38 °C/decade for RCP 8.5 for a set of Swiss lakes at varying altitudes. This is consistent with the observational study by Dokulil et al. (2021) reporting higher temperature increases in the alpine region compared to other European lakes.

Our model results for the years 2006–2020 and for the reference period 1970–2005 tend to underestimate the observational trends in surface temperature, hence the model predictions should be interpreted as conservative estimate. Although FLake performs poorly at the bottom, the predictions for bottom temperatures are in agreement with previous modelling studies of lakes in Northern Germany (Shatwell et al. 2019), Switzerland (Råman Vinnå et al. 2021) and globally (Golub et al. 2022) as well as with global observations (Pilla et al. 2020). Bottom warming was much lower or—in the less extreme emission scenario—not observed at all. Temperatures at the lake bottom depend on the temperature during spring mixing which occurs earlier but at similar temperatures under warming climate conditions (Bartosiewicz et al. 2019). In addition, the duration of the stratification process plays an important role for the deepwater temperature but is difficult to predict. If, for example, an existing but weak stratification is mixed up again during a storm, the hypolimnion temperature increases. It is possible that warmer spring temperatures favour a quick stratification process with comparably lower hypolimnion temperatures.

In combination with overall higher temperatures, the increasing temperature gradient between surface and bottom causes an extension of summer stratification in dimictic lakes accompanied by a shortening of the winter stratification (Fig. 4e, f). Compared to the first decade 2005–2015, the difference between bottom and surface temperatures increased by approximately 37% for RCP 8.5 until the end of the century. In parallel, the thermal expansion coefficient at the thermocline increased at a similar rate, by 46%, due to overall higher temperatures. This implies that both, total temperature increases and higher temperature gradients, have comparable effects on the increasing summer stratification. The relative importance of the two effects was comparable in the other two emission scenarios. Similar results were found by Schwefel et al. (2016) for Lake Geneva.

An earlier onset of stratification (19 days) and a later mixing in fall (also 19 days) results in a combined prolongation of the stratified period by 38 days. The trend in the onset of stratification was found universally in all lakes. In contrast, the end of stratification changed more strongly in deep lakes. This could be a result of different mixing mechanisms in shallow and deep lakes. All lakes experience a gradual deepening of the mixed layer during summer which accelerates in autumn. Therefore, deep lakes are mixing later in the year because the mixed layer needs more time to reach the deepest point. While the main mechanism for mixing during summer is wind stress, convection (Bouffard and Wüest 2019) starts to play a more important role during the end of the year. In all climate scenarios, average wind speed showed no significant trend (except for Lake Schaalsee in autumn for RCP 2.6 and Lake Titisee in summer and winter for RCP 8.5), hence the prolongation of the stratified period in shallow lakes is caused mainly by a stronger summer stratification which requires more energy to be overcome. However, air temperature was increasing consistently in all emission scenarios resulting in weaker convection during fall and winter and therefore acting as an additional driver for the prolongation of the stratified period in deep lakes. The overall change in the stratified period of 38 days is very close to the 33 days found by Woolway et al. (2021) based on a global dataset also using simulations performed with FLake. In contrast to our findings, their study reports a higher relevance of an earlier stratification start (22 earlier start and 11 days later end compared to 19 days each in our study). These differences could be caused by a larger number of shallow lakes in their dataset, although interpretations have to be handled with care since the end of stratification is less reliably predicted by Flake compared to the onset. Our findings highlight that the response of lakes to climate change can depend on locally varying lake morphometry and that global or regional trends must be handled with care if transferred to local conditions.

### Impact on the deepwater oxygen concentrations

Oxygen in the hypolimnion is crucial for higher forms of life and to suppress the release of phosphorus or reduced substances such as Mn^2+^ or Fe^2+^ from the sediments. In dimictic or monomictic lakes, climate change could affect DO budgets either by increasing mineralization rates due to higher temperatures (see e. g. Gudasz et al. 2010 for sediment mineralization rates), or by delayed resupply of DO into the hypolimnion caused by an extension of the stratification period (Jane et al. 2023). The model by Nkwalale et al. (2024) allowed us to estimate averaged hypolimnetic DO concentrations as a function of lake stratification, initial DO concentration in spring, trophic state and hypolimnion temperatures.

Although our DO model provides a practical tool to assess long-term trends of hypolimnetic DO concentrations based on very limited input parameters, it has two fundamental limitations. Firstly, it does not resolve vertical gradients. In reality, DO depletion is increasing with depth leading to a gradual build-up of anoxia from the bottom over the course of the summer (Schwefel et al. 2018; LaBrie et al. 2023). This implies that the anoxic or hypoxic layer in the deep water thickens with increasing stratification duration and decreasing average DO concentrations even if average DO concentrations remain above the threshold for hypoxia (Jane et al. 2023). Secondly, the model is based on statistical relationships between trophic state and DO depletion. The statistically found oxygen depletion rates were derived based on observational data under climate conditions typical for the recent past and often in lakes which were experiencing man-made eutrophication (Nkwalale et al. 2024). However, long-term observations show that primary production and in turn DO depletion does not necessarily react linearly to changes in nutrient concentrations. Often, productivity and DO depletion does not decrease directly to pre-eutrophic conditions in response to nutrient limitations (Schwefel et al. 2018; Anneville et al. 2002; Merz et al. 2023), and reduced substances diffusing out of older sediment layers from a more eutrophic past can contribute considerably to the DO depletion (Matzinger et al. 2010; Steinsberger et al. 2021). Hence, scenarios with changing trophic states have to be handled with care. Nevertheless, legacy effects will be probably minimized if changes in trophic state happen over a long period of time as it is assumed in our simulation over almost a full century.

If nutrient concentrations do not change, possible changes in DO concentrations could be caused by (1) insufficient DO supply during the mixing period, (2) changes in hypolimnion temperatures, or (3) an extended stratification period. Our simulations predict that all lakes continue to mix completely at least once per year, hypolimnetic oxygen concentrations can still be considered to be fully reset after spring mixing (Pilla et al. 2023) and bottom temperatures warmed much less than surface temperatures. Therefore, the prolongation of the stratified period was the main driver for decreasing DO levels. This is consistent with global observations (Jane et al. 2023) and is also predicted in the shallow Mississippi Lake by Yaghouti et al. (2023) using the biogeochemical model CE-QUAL-W2 with a much higher complexity than our oxygen depletion model.

On average, DO concentrations at the end of the stratified period decreased by 1.2 mg L^−1^ in 2086–2099 compared to 2006–2020 in the most pessimistic scenario RCP 8.5. As shown in Fig. 5, temporally stratified lakes of all trophic states are affected by these changes. Losses were higher in eutrophic or mesotrophic deep lakes in which the period of summer stratification extended the most and rates of DO depletion were high (Fig. 6a). In oligotrophic Lake Titisee, DO concentrations at the end of summer stagnation decreased by only 0.7–0.6 mg L^−1^ until the end of the century. On the other hand, in the eutrophic Lake Arendsee, average hypolimnetic DO concentrations decreased by 1.9 mg L^−1^ on average and often dropped to zero before the stratification period ended, indicating complete anoxic conditions with severe consequences for the ecosystem. Decreases in DO concentration were lower in the emission scenarios RCP 4.5 (− 0.6 mg L^−1^ in average) and RCP 2.6 (− 0.2 mg L^−1^) and even in Lake Arendsee, which consistently experienced the lowest DO concentrations, complete anoxic conditions during the end of summer did not occur. Given that the model predicts only volume-averaged oxygen concentrations, concentrations above 0 mg L^−1^ do not exclude the possibility of anoxic conditions in the deep layers of the hypolimnion which experience typically higher oxygen depletion due to larger sediment-to water volume ratios (Livingstone and Imboden 1996). Decreasing average DO concentrations therefore imply increasing fractions of the hypolimnion being affected by anoxic conditions.

To maintain a healthy lake ecosystem, it is important to quantify the relative importance of global effects caused by climate warming in relation to local management efforts. Therefore, we assumed in scenario B a change in trophic states from eutrophic to mesotrophic (or from mesotrophic to oligotrophic, respectively) e. g. due to decreasing nutrient inputs from the catchment. In all lakes affected by changes in trophic state, the resulting lower DO depletion rate exceeded the negative consequences of warming even for the most pessimistic climate scenario. Averaged over all lakes, mean hypolimnetic DO concentrations at the end of summer stratification increased by at least 1.1 mg L^−1^ for the most pessimistic emission scenario RCP 8.5. These results highlight that metabolic processes and their changes in response to altering nutrient concentrations remain the key driver for changes in the oxygen budget (Ladwig et al. 2022). As a result, negative effects of climate change can be mitigated by lake management measures. Similar results were obtained by Matzinger et al. (2007), who studied the combined effect of eutrophication and climate change on Lake Ohrid using a coupled biogeochemical and hydrodynamic model. They assessed that a reduction in phosphorus loads by 50% would be necessary to mitigate the effects of an average temperature increase of + 0.4 °C/decade (comparable to emission scenario RCP 8.5 in Germany). While DO conditions in reservoirs can be improved by additional measures such as varying withdrawal strategies (Feldbauer et al. 2020; Mi et al. 2020), limiting nutrient loads from the catchment seems to be one of the few tools to mitigate negative effects of climate change in natural lakes in light of a global decrease in hypolimnetic DO concentrations (Jane et al. 2021, 2023). Our results indicate that these measures have the potential to be highly effective and that their positive effects can exceed the negative impacts of climate change.

The findings of this study support the idea that climate change exhibits similar patterns to those of eutrophication described by (Moss et al. 2011) with hypolimnetic DO concentrations expected to decline further if current trophic states persist. However, we stress the importance of reducing nutrient loading in lake catchments as a means of lowering trophic state to mitigate the adverse effects of climate warming (Jeppesen et al. 2017) amidst declining hypolimnetic DO concentrations globally (Jane et al. 2021, 2023). However, this climate adaptation mechanism takes time to yield positive results, thus, in the face of a climate warming mediated crisis requiring immediate action, other faster mitigation measures should be considered. For instance, DO conditions can be improved in the short-term by implementing varying withdrawal strategies in reservoirs (Feldbauer et al. 2020; Mi et al. 2020) or by installing aeration/oxygenation systems in lakes facing persistent hypoxia/anoxia (Beutel and Horne 1999; Gantzer et al. 2009; Beutel et al. 2014). Another avenue for climate adaptation in management strategies involves broadscale modelling efforts, encompassing both the scale presented in this study and short-term forecasting approaches (Carey et al. 2022) to establish early warning systems against ecosystem declines.

## Conclusion

We investigated changes in thermal structure and dissolved oxygen (DO) budgets in a series of German lakes in response to climate change using long-term monitoring data between 1990 and 2020, the one-dimensional model FLake for the years 2006–2099, and an coupled DO model (Nkwalale et al. 2024). Our main findings were:

- Averaged over 46 lakes, surface temperatures increased by 0.5 °C/decade between 1990 and 2020. Numerical modelling of 12 lakes for the years 2006–2099 predicts further increases of 0.30 ± 0.08 °C for emission scenario RCP 8.5, 0.11 ± 0.05 °C for RCP 4.5, and 0.04 ± 0.01 °C for RCP 2.6. Increases were homogenous between lakes but varied considerably depending on the chosen GCM/RCM-combination. In contrast to surface temperatures, trends in bottom temperatures were lower and only significant for climate scenario RCP 4.5 and RCP 8.5 (+ 0.02 and + 0.05 °C/decade, respectively).
- In response to these temperature changes, the duration of summer stratification is predicted to increase by 38 days for emission scenario RCP 8.5 (+ 22 days for RCP 4.5; + 13 days for RCP 2.6). The change was most pronounced in deep lakes. In parallel, winter stratification shortened, and deeper lakes experienced a regime shift from a dimictic to a monomictic mixing regime.
- According to the DO model, the average DO concentration at the end of summer decreases by 1.2 mg L^−1^ (RCP 8.5), 0.6 mg L^−1^ (RCP 4.5), or 0.2 mg L^−1^ (RCP 2.6) if trophic states remain unchanged. Higher changes are expected in deep and eutrophic lakes. Here, the whole hypolimnion could become anoxic at the end of summer with severe consequences for the entire ecosystem. If the trophic state of eutrophic and mesotrophic lakes would improve, e.g., due to limited nutrient inputs, the resulting decreasing DO depletion rates would outweigh the effect of climate change and average DO concentrations would increase even under the most pessimistic emission scenario RCP 8.5 and be more than 1 mg L^−1^ on average. This finding indicates that local measures can mitigate effects of global warming, at least for the problem of hypolimnetic deoxygenation.

## Supplementary Information

Below is the link to the electronic supplementary material.

Supplementary file1 (PDF 1388 kb)
