# Supplementary material for: Temperatures and hypolimnetic oxygen in German lakes: Observations, future trends and adaptation potential
Source: Ambio. 2024 Jul 5;54(3):428–47. doi: 10.1007/s13280-024-02046-z (PMC11780045; doi:10.1007/s13280-024-02046-z)
Supplement: Supplementary file 1 — Supplementary file1 (PDF 1388 kb) [file 13280_2024_2046_MOESM1_ESM.pdf]

Ambio
Supplementary Information

This supplementary information has not been peer reviewed.

Title: **Temperatures and hypolimnetic oxygen in German lakes – observations, future trends, and adaptation potential.**

Table S 1: Root mean square error (RMSE), calibration between modelled and observed values, bias ($T_{observed}$-T_{model}), and Nash-Sutcliffe-Efficiency (NSE) of the numerical model FLake during the calibration period and the full period of the validation in the 12 simulated lakes. In the polymictic Lake Müggelsee, no distinction between surface and bottom water was made. In Lake Plöner See, Tiefwarensee, Schaalsee, and Tollensesee measurement periods were too short to separate between calibration and validation period.

Lake	Calibration RMSE [°C]		Calibration Correlation [-]		Calibration bias [°C]		Calibration NSE	
	Surface	Bottom	Surface	Bottom	Surface	Bottom	Surface	Bottom
Arendsee	0.93	0.75	0.99	0.86	-0.05	-0.43	>0.99	0.65
Breiter Luzin	1.06	0.75	0.99	0.32	-0.39	-0.59	0.97	-2.5
Plöner See	1.25	0.23	0.98	0.75	-0.46	0.26	0.94	-0.95
Laacher See	1.84	0.52	0.97	0.34	-1.53	0.06	0.93	-0.28
Titisee	1.13	1.13	0.97	0.72	-0.35	-0.73	0.97	-5
Tegeler See	1.63	2.71	0.99	0.91	1.44	2.07	0.92	0.06
Schm. Luzin	0.84	0.39	0.99	0.90	0.46	0.12	0.97	-15
Haussee	1.46	0.80	0.99	0.95	0.99	-0.01	0.95	0.86
Müggelsee	0.85	n.a.	0.99	n.a.	0.67	n.a.	0.99	n.a.
Tiefwarensee	1.06	2.07	0.99	0.30	-0.73	1.84	0.96	-1.6
Schaalsee	2.13	0.72	0.95	0.68	0.56	0.63	0.90	-1.1
Tollensesee	1.19	2.25	0.98	0.82	0.01	1.78	0.96	-1.2

Lake	Validation RMSE [°C]		Validation Correlation [-]		Validation bias [°C]		Validation NSE	
	Surface	Bottom	Surface	Bottom	Surface	Bottom	Surface	Bottom
Arendsee	1.45	0.75	0.97	0.60	-0.33	-0.63	>0.99	-0.27
Breiter Luzin	1.69	0.73	0.98	0.29	-0.45	-0.15	0.99	-0.80
Plöner See	n.a.	n.a.	n.a.	n.a.	n.a.	n.a.	n.a.	n.a.
Laacher See	1.49	1.00	0.98	0.76	-1.11	-0.46	0.91	-0.70
Titisee	1.32	0.99	0.99	0.42	-0.71	-.31	0.92	-3
Tegeler See	1.74	2.50	0.98	0.80	1.19	2.82	0.94	-0.34
Schm. Luzin	n.a.	n.a.	n.a.	n.a.	n.a.	n.a.	n.a.	n.a.
Haussee	1.69	1.96	0.98	0.74	0.89	1.20	0.94	0.19
Müggelsee	1.39	n.a.	0.99	n.a.	0.99	n.a	0.97	n.a.
Tiefwarensee	n.a.	n.a.	n.a.	n.a.	n.a.	n.a.	n.a.	n.a.
Schaalsee	n.a.	n.a.	n.a.	n.a.	n.a.	n.a.	n.a.	n.a.
Tollensesee	n.a.	n.a.	n.a.	n.a.	n.a.	n.a.	n.a.	n.a.

Table S 2: Overview of the combinations of global circulation models and regional downscaling models used in the DWD-Kernensemble.

RCP 2.6		RCP 4.5		RCP 8.5	
GCM	RCM	GCM	RCM	GCM	RCM
MOHC-HadGEM2-ES (r1)	KNMI-RACMO22E	ICHEC-EC-EARTH (r1)	KNMI-RACMO22E	ICHEC-EC-EARTH (r1)	KNMI-RACMO22E
ICHEC-EC-EARTH (r12)	KNMI-RACMO22E	ICHEC-EC-EARTH (r12)	KNMI-RACMO22E	CCCma-CanESM2 (r1)	CLMcom-CCLM4-8-17
ICHEC-EC-EARTH (r12)	CLMcom-CCLM4-8-17	ICHEC-EC-EARTH (r12)	SMHI-RCA4	MOHC-HadGEM-ES (r1)	CLMcom-CCLM4-8-17
MIROC-MIROC5 (r1)	CLMcom-CCLM4-8-17	MOHC-HadGEM-ES (r1)	CLMcom-CCLM4-8-17	MIROC-MIROC5(r1)	GERICS-REMO2015
MPI-M-MPI-ESM-LR (r2)	MPI-CSC-REMO2009	MPI-M-MPI-ESM-LR (r1)	MPI-CSC-REMO2009	MPI-M-MPI-ESM-LR (r1)	UHOH-WRF361H
		MPI-M-MPI-ESM-LR (r2)	MPI-CSC-REMO2009	MPI-M-MPI-ESM-LR (r2)	MPI-CSC-REMO2009

Table S 3: Coefficients of a multiple linear model for the bottom water temperature as function of air temperature, lake depth and lake surface area.

Coefficient	Estimate	Standard Error	p-Value
Intercept	5,5861	0,0437	<1e-10
Air Temperature [°C]	0,0919	0,0031	<1e-10
Lake Depth [m]	-0,0168	0,0004	<1e-10
Lake Area [km2]	0,0007	0,00001	<1e-10

Table S 4: Coefficients of a multiple linear model for the oxygen concentration at the bottom of stratified lakes (below 80% of the maximal depth) as function of water temperature, phosphorus concentration and lake depth.

Coefficient	Estimate	Standard Error	p-Value
Intercept	4,95	0,36	<1e-10
Water Temperature [°C]	-0,382	0,049	<1e-10
Total phosphorus [mg/m^3]	-9,38	0,63	<1e-10
Lake Depth [m]	0,0335	0,0020	<1e-10

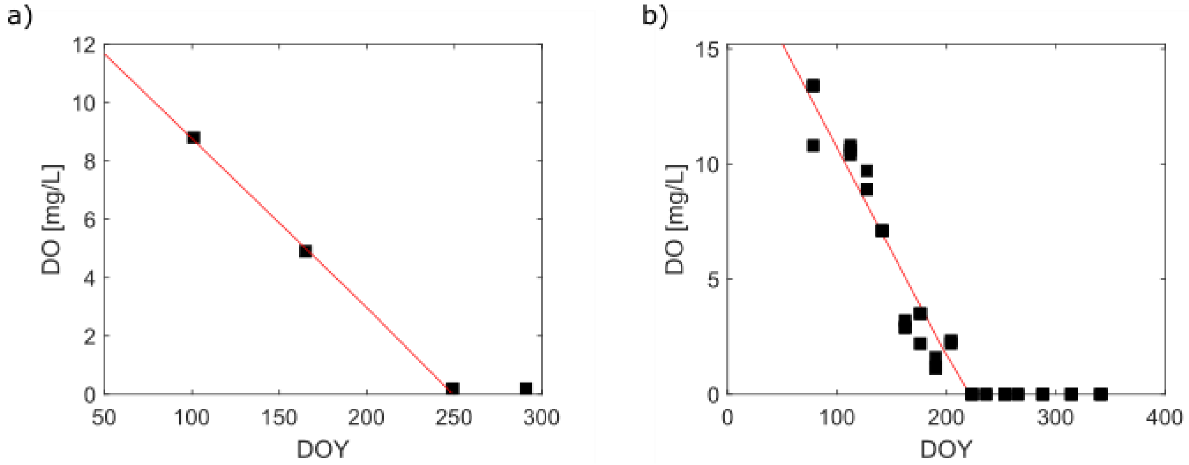

Figure S 1: Example of linear fits of bottom DO concentrations during summer. a) Alpsee 2014, b) Breiter Luzin 2015.

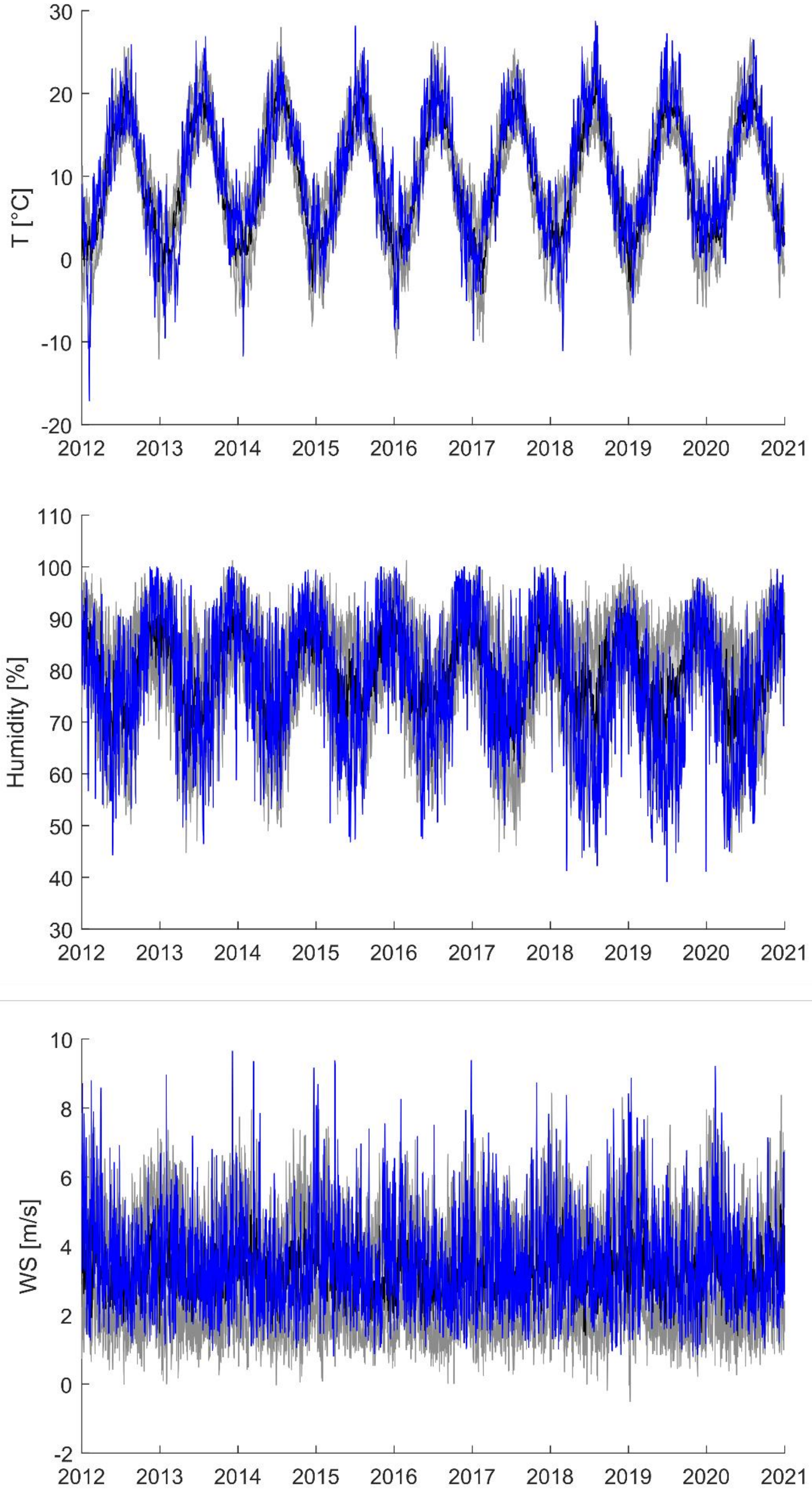

Figure S 2: Comparison of data from the meteorological station Seehausen close to Lake Arendsee (blue) with the predictions of the 6 combinations of global circulation models and regional downscaling models (GCM/RCM) of the DWD-Kernensemble assuming the IPCC emission scenario RCP 8.5 (black, standard deviation indicated as gray area).

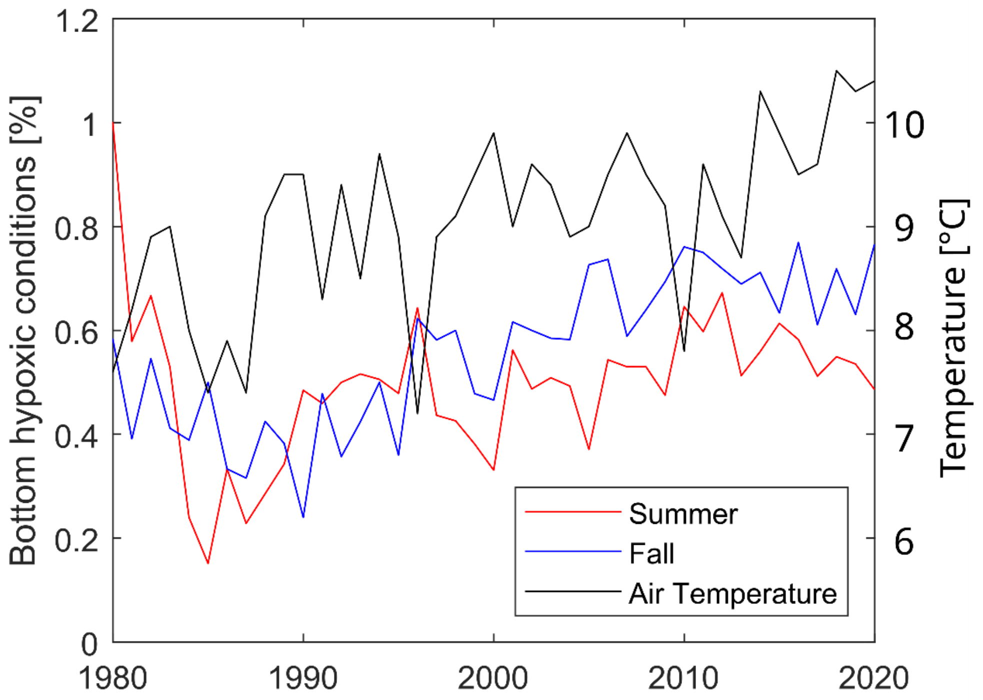

Figure S 3: Mean annual air temperatures in Germany (black, right axis) and fraction of lakes experiencing hypoxic or anoxic conditions (O2 < 2 mg/L, left axis) in Fall and Summer.

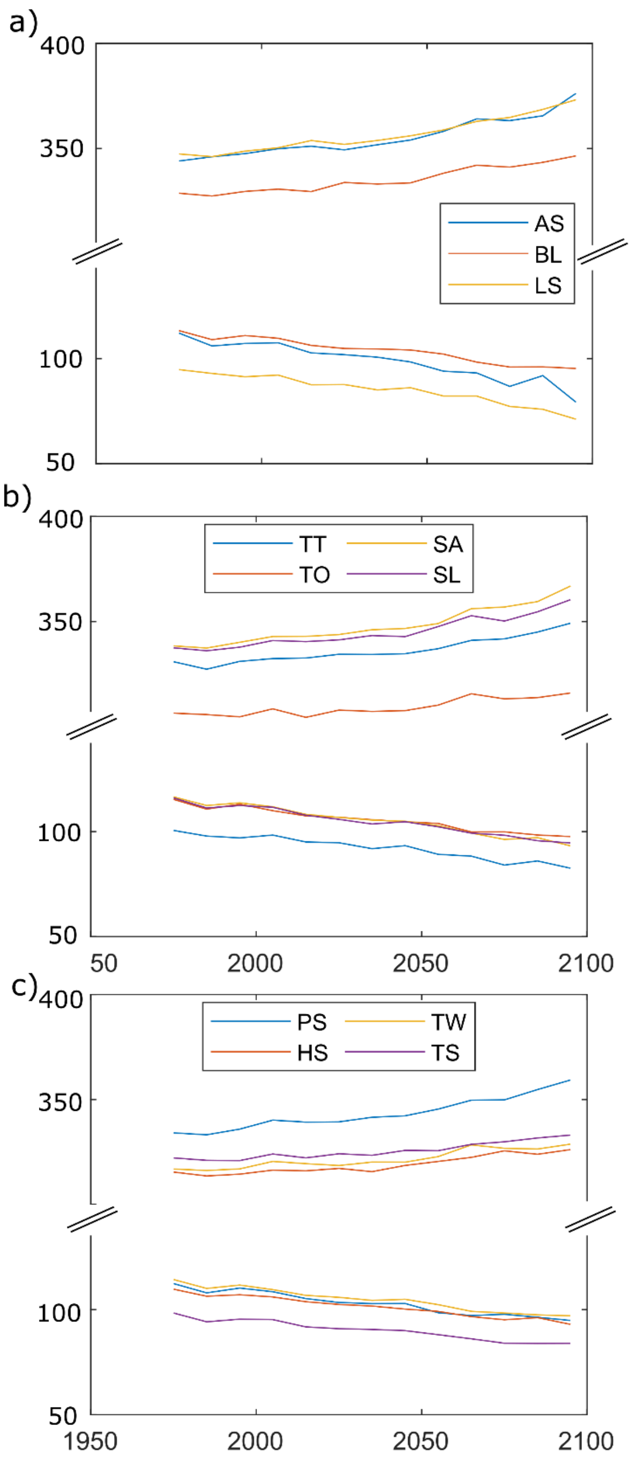

Figure S 4: Begin and end of the stratified period during summer. a) Arendsee (AS), Breiter Luzin (BL), Laacher See (LS); b) Titisee (TT), Schaalsee (SA), Tollensesee (TO), and Schmaler Luzin (SL); c) Plöner See (PS), Tiefwarensee (TW), Haussee (HS), Tegeler See (TS).

Text S1: Matlab-Function “Nkwalale_et_al_O2_model.m”. For details, see Nkwalale, L., R. Schwefel, M. Yaghouti, and K. Rinke. 2024. A simple model for predicting oxygen depletion of lakes under climate change. Inland Waters. doi:10.1080/20442041.2024.2306113.

```
function [DOend,Lake,Year] = Nkwalale_et_al_O2_model(indata,altitude);
%NKWALALE_ET_AL_O2_MODEL For details, see Nkwalale, L., R. Schwefel, M. Yaghouti,
% and K. Rinke. 2024. A simple model for predicting
% oxygen depletion of lakes under climate change. Inland Waters.
% doi:10.1080/20442041.2024.2306113.
% input:
% altitude: lake altitude [m]
% indata: csv with Lake Name, Year, Stratification duration [days], and
%Temperature [°C] including one (arbitrary) header line.
%
% example:
% Lake, Year, Stratification [days], Temperature [°C]
% Arendsee, 2006, 234, 4.19
% Arendsee, 2007, 237, 5.05
% ...
%
% output:
% Lake: Lake Name
% Year: Year of calculation
% DOend: Oxygen concenration at end of stratified period [mg/L],
% column 1: assuming oligotrophic conditions, column 2: mesotrophic conditions,
% column 3: eutrophic conditions
%
% output is also written to a file called Output_O2.csv

    AllData = readtable(indata,'NumHeaderLines',1);
    Lake = AllData.Var1;
    Year = AllData.Var2;
    S = AllData.Var3;
    T = AllData.Var4;

    % constants according to Nkwalale et al. 2024 (median values for depletion
    %rates)

    DEP_o = 0.0209;DEP_m = 0.033; DEP_e = 0.0412; k = 2.3^.1;

    %Temperature in Kelvin:
    TK = T+273.1524;

    % partial pressure based on altitude assuming scale height of 8400 m:
    DOpart = (20.95/100)*exp(-altitude/8400);

    %initial DO assuming 100% saturation (see Nkwalale et al. 2024):
    DO_ini =exp(58.3877+85.8079.*(100./TK)+23.8439.*log(TK/100)).*(986.9/22391)...

       *1000.*DOpart.*32;

    % depletion rate depending on temperature and trophic state:
    DEP(:,1) = DEP_o.*k.^(T-4);
    DEP(:,2) = DEP_m.*k.^(T-4);
    DEP(:,3) = DEP_e.*k.^(T-4);

    % DO at the end of stratification
    DOend(:,1) = DO_ini-DEP(:,1).*S;
    DOend(:,2) = DO_ini-DEP(:,2).*S;
    DOend(:,3) = DO_ini-DEP(:,3).*S;
```

```
    % avoid negative DO. Remove this line if you want to allow negative DO
    % concentrations (e.g., as proxy for reduced substances diffusing
    % out of the sediment).
    DOend(DOend<0) = 0;

    % write result to file
    fid = fopen('Output_O2.csv', 'wt' );
    fprintf(fid ...

       ,'Lake, Year, Stratification [days], DO_end_oligotrophic
       [mg/L],DO_end_mesotrophic [mg/L],DO_end_eutrophic [mg/L]\n');
    for i = 1:length(T)
        fprintf(fid,'%s, %d, %.2f, %.2f, %.2f \n', ...

         Lake{i},Year(i),DOend(i,1),DOend(i,2),DOend(i,3));
    end
    fclose(fid);

end
```
